# L-carnitine reduces acute lung injury via mitochondria modulation and inflammation control in pulmonary macrophages

**DOI:** 10.1590/1414-431X2023e12830

**Published:** 2023-10-20

**Authors:** Dandan Wu, Haiyan He, Jinliang Chen, Sumei Yao, Haiqin Xie, Wenyan Jiang, Xuedong Lv, Wei Gao, Linlin Meng, Xin Yao

**Affiliations:** 1 Department of Respiratory and Critical Care Medicine, The First Affiliated Hospital of Nanjing Medical University, Guangzhou, Nanjing, China; 2 Department of Respiratory and Critical Care Medicine, The Second Affiliated Hospital of Nantong University, North Haierxiang, Nantong, China; 3 Department of Pulmonary and Critical Care Medicine, Shanghai East Hospital, Tongji University School of Medicine, Jimo, Shanghai, China

**Keywords:** Acute lung injury, Macrophages, L-carnitine, Lipid accumulation, Anti-inflammation

## Abstract

Acute lung injury (ALI) or acute respiratory distress syndrome (ARDS) is a critical respiratory syndrome with limited effective interventions. Lung macrophages play a critical role in the pathogenesis of abnormal inflammatory response in the syndrome. Recently, impaired fatty acid oxidation (FAO), one of the key lipid metabolic signalings, was found to participate in the onset and development of various lung diseases, including ALI/ARDS. Lipid/fatty acid contents within mouse lungs were quantified using the Oil Red O staining. The protective effect of FAO activator L-carnitine (Lca, 50, 500, or 5 mg/mL) was evaluated by cell counting kit 8 (CCK-8) assay, real-time quantitative PCR (qPCR), ELISA, immunoblotting, fluorescence imaging, and fluorescence plate reader detection in lipopolysaccharide (LPS) (100 ng/mL)-stimulated THP-1-derived macrophages. The *in vivo* efficacy of Lca (300 mg/kg) was determined in a 10 mg/kg LPS-induced ALI mouse model. We found for the first time that lipid accumulation in pulmonary macrophages was significantly increased in a classical ALI murine model, which indicated disrupted FAO induced by LPS. Lca showed potent anti-inflammatory and antioxidative effects on THP-1 derived macrophages upon LPS stimulation. Mechanistically, Lca was able to maintain FAO, mitochondrial activity, and ameliorate mitochondrial dynamics. In the LPS-induced ALI mouse model, we further discovered that Lca inhibited neutrophilic inflammation and decreased diffuse damage, which might be due to the preservation of mitochondrial homeostasis. These results broadened our understanding of ALI/ARDS pathogenesis and provided a promising drug candidate for this syndrome.

## Introduction

Acute lung injury (ALI), or its severe form acute respiratory distress syndrome (ARDS), is a common life-threatening condition among critically ill patients, characterized by overwhelming lung inflammation and diffuse alveolar damage (1). Although there have been great advances in critical care medicine and organ supportive therapies, ALI/ARDS remains a prominent health, social, and economic problem, with very few pharmacotherapies and a high mortality rate (2). Therefore, novel approaches to control excessive inflammation and intensive damage in ALI/ARDS are urgently needed.

Among the immune cells involved in the pathogenesis of ALI/ARDS, lung macrophages are crucial to the initiation and progression of the overwhelming pulmonary inflammation and have the potential to become a therapeutic target (1,3). In the early stage of the syndrome, macrophages can be rapidly activated by microbial stimuli, which subsequently boost the detrimental inflammation by producing multiple cytokines and recruiting excess inflammatory cells to the lung. Mechanistically, the activation of nucleotide-binding oligomerization domain, leucine-rich repeat and pyrin domain-containing 3 (NLRP3)‐inflammasome, and the secretion of pro-inflammatory interleukin-1β (IL-1β) are critical in the development of lipopolysaccharide (LPS)-induced ALI (4). Thus, modulating inflammatory reactions of pulmonary macrophages represents a promising strategy for the treatment of ALI/ARDS.

Once simply regarded as the reserves of metabolic energy, lipid metabolism is now considered to take part in various physiological and pathological processes (5). As one of the key lipid metabolic signalings, fatty acid oxidation (FAO) mainly occurs in mitochondria and regulates important immune responses, thus exhibiting great clinical significance. Although the lung is seldom viewed as a metabolic organ, active lipid metabolism often occurs, especially in the alveolar area. Metabolic disorder of the lipids can induce inflammatory over-reaction, excess oxidative stress, and tissue injury, which may contribute to the onset and development of various lung diseases, such as ALI/ARDS (6).

In an LPS-induced animal model of ALI, impaired FAO of alveolar epithelial cells was demonstrated to participate in the acute inflammation and tissue damage, which was likely attributed to the defective mitochondrial bioenergenesis (7). In another hyperoxia-mediated lung injury model of newborn mice, pharmaceutical inhibition of FAO in endothelial cells aggravated their apoptosis while enhancing FAO with L-carnitine (Lca) alleviated the lung injury in neonates (8). Furthermore, defective FAO in the chronic activated alveolar macrophages by multi-walled carbon nanotube (MWCNT) contributed to the mitochondrial dysfunction and pulmonary granulomatous inflammation (9). Previously, Lca has been shown to reduce lung injury due to obstructive jaundice (10) and potassium dichromate (11) in rat models; however, the effect of Lca in the LPS-induced ALI model and the detailed mechanisms deserve further investigation. In this study, we evaluated the effect of Lca, the mitochondrial FAO activator, on inflammatory cytokines expression, intracellular and mitochondrial reactive oxygen species (ROS) production, and mitochondrial dynamics triggered by LPS in THP-1 derived macrophages. Moreover, we investigated the anti-inflammatory efficacy of Lca and its possible mechanisms in the murine model of ALI/ARDS.

## Material and Methods

### Cell culture

THP-1 cells, the human monocytic cell line, were cultured in RPMI-1640 medium (Hyclone, USA) supplemented with 2 mM L-glutamine, 1 mM sodium pyruvate (Gibco, USA), and 10% fetal bovine serum (FBS) (Gibco). Cells were differentiated into macrophage-like cells by stimulating with 50 ng/mL phorbol-12-myristate-13-acetate (PMA, Sigma Aldrich, P8139, USA). After 24 h, the adherent cells were washed with phosphate buffered saline (PBS) three times followed by resting for 48 h before further experiments.

### Cell viability detection

THP-1 cell-derived macrophages were treated with Lca (50, 500, or 5 mg/mL) (12) in the absence or presence of LPS (100 ng/mL, InvivoGen, USA) (13) stimulation for 24 h. Cell viability was then determined by cell counting kit 8 (CCK-8) assay (Dojindo Laboratories, CK04, Japan). The CCK-8 reagent (10 μL/well) was directly added into the 96-well plate and incubated for 1.5 h at 37°C. The absorbance at 450 nm was measured using a SpectraMax iD3 microplate reader (Molecular Devices, USA).

### Cytokine assay

THP-1 cells were seeded onto a 96-well plate (1×10^5^ cells/well) and 12-well plate (5×10^5^ cells/well), followed by differentiating into adherent macrophages. Then cells were exposed to LPS alone or combined with Lca at different concentrations for 24 and 6 h, respectively. The levels of human interleukin-6 (IL-6) (MultiSciences Biotech, EK106/2-96, China), tumor necrosis factor-α (TNF-α) (MultiSciences Biotech, EK182-96), and IL-1β (MultiSciences Biotech, EK101B-96) in the cell supernatants of the 96-well plate were detected by ELISA kits according to the manufacturer's instructions.

The mRNA expression of cytokines in cells in the 12-well plate was determined by real-time quantitative PCR (qPCR). Total RNA was extracted by TRIzol reagent (Invitrogen, 15596026, USA), followed by converting to cDNA using the PrimeScript™ Reverse Transcriptase Reagent Kit (Takara, RR036A, Japan). The SYBR Green qPCR Mix and ABI ViiATM 7 System were applied for the qPCR assay. The specific primers for human IL-6, TNF-α, IL-17A, peroxisome-proliferator activated receptor (PPAR), liver X receptor (LXR), adenosine triphosphate-binding cassette transporter A1 (ABCA1), and β-actin were purchased from BioTNT (China) and their sequences are as follows: Human IL-6: Forward: ACTCACCTCTTCAGAACGAATTG, Reverse: CCATCTTTGGAAGGTTCAGGTTG; Human TNF-α: Forward: CCTCTCTCTAATCAGCCCTCTG, Reverse: GAGGACCTGGGAGTAGATGAG; Human IL-17A: Forward: CCCCCGGACTGTGATGGTCAAC, Reverse: GCGGCACTTTGCCTCCCAGAT; Human PPAR: Forward: CGTGCTTCCTGCTTCATAGATAAG, Reverse: GTGGTAGCGCTGGTCTAC; Human LXR: Forward: AGAAGAACAGATCCGCCTGAAG, Reverse: GGCAAGGATGTGGCATGAG; Human ABCA1: Forward: GCAGCAGAGCGAGTACTTCGTT, Reverse: CAAGACTATGCAGCAATGTTTTTGT; Human β-actin: Forward: CCTGGCACCCAGCACAAT, Reverse: GCCGATCCACACGGAGTACT.

### Intracellular and mitochondrial reactive oxygen species (ROS) assay

THP-1 cell-derived macrophages were plated onto 12-well covered glass-bottom chambers and underwent different treatments. After 24 h, cells were incubated with MitoTracker Red CMXRos (MTR) staining solution (Invitrogen, M7512). The cells were then observed under a fluorescence microscope (Olympus CKX53SF, Japan), with the fluorescence intensity representing the integrity of mitochondria.

For mitochondrial ROS assay, cells seeded onto a 96-well black plate were co-cultured with MitoSOX™ Red probe (Invitrogen, M36008) in the dark for 10 min at 37°C. The fluorescence of MitoSOX at 510/580 nm was recorded under the fluorescence plate reader (Molecular Devices).

As for intracellular ROS detection, THP-1 cell-derived macrophages were plated onto a 96-well black plate and incubated with 10 μM 2',7'-dichlorodihydrofluorescein diacetate (DCFH-DA, Sigma-Aldrich, 35845) in the dark for 15 min at 37°C. Cells were then washed with serum-free RPMI-1640 three times, followed by detecting the intracellular ROS at 488/525 nm under a Flexstation3^®^ (Molecular Devices, USA) fluorescence plate reader.

### Immunoblotting analysis

Whole cell lysates and their concentration quantification were conducted based on a previous study (14). Equal amounts of protein were resolved on 10% sodium dodecyl sulfate-polyacrylamide gels under 120 V and transferred to the PVDF membranes (Millipore, USA). Nonspecific sites on the membranes were blocked with tris-buffered saline (TBS) that contained 5% nonfat milk and 0.1% Tween 20 (TBST, Beyotime, China) for 2 h. Each blot was incubated with primary antibodies against phosphorylated p65 (Cell Signaling Technology, 3033, USA), p65 (Cell Signaling Technology, 8242), NLRP3 (Cell Signaling Technology, 15101), β-actin (Cell Signaling Technology, 3700), optic atrophy 1 (OPA1, Cell Signaling Technology, 80471), mitofusions 2 (MFN2, Cell Signaling Technology, 9482), dynamin-related protein 1 (DRP1, Cell Signaling Technology, 8570), and Fission1 (FIS1, Zenbio, 505821, China) overnight at 4°C. After rinsing with TBST 3 times, blots were incubated with horseradish peroxidase (HRP)-conjugated secondary antibodies (Cell Signaling Technology, 7074, 7076) for 1.5 h. The signal of each protein was imaged on a Tanon-4200 image analyzer (Tanon Biotechnology, China) via ECL chemiluminescence method and analyzed by ImageJ software (NIH, USA).

### Animal study approval

All procedures were conducted based on the guidelines of Shanghai Committee for the Accreditation of Laboratory Animals and approved by the Laboratory Animal Research Center Review Board of Tongji University (Permit Number: TJBB03721106; China). Murine surgeries were performed under sodium pentobarbital anesthesia, and all efforts were made to minimize animal suffering.

### LPS-induced ALI murine model

Six to eight-week-old male C57BL/6 mice were obtained from Model Animal Research Center of Nanjing University (China). After being anesthetized with chloral hydrate (5%, 0.08 mL/10 g) through an intraperitoneal injection, Lca (300 mg/kg) (15) or PBS were intratracheally injected 2 h before LPS (10 mg/kg) (13) or PBS intranasal administration. The mice were sacrificed 24 h after LPS challenge for further evaluation.

### Bronchoalveolar lavage fluid (BALF) collection and analysis

After anesthesia, 0.8 mL of ice-cold PBS was injected slowly into the mouse airway and the retrieved fluid was carefully collected as the BALF. BALF total inflammatory cells were obtained after centrifugation (350 g for 5 min at 4°C) and counted by a hemocytometer with trypan blue dye test. Cells were then fixed on slides and stained with Wright-Giemsa. Cell differential count was performed under a microscope in at least 200 cells/sample. Moreover, BALF supernatants were analyzed for cytokine, chemokine, and total protein levels. Mouse keratinocyte chemoattractant (KC) (MultiSciences Biotech, EK296/2-96, China), IL-6 (MultiSciences Biotech, EK206/3-96), TNF-α (MultiSciences Biotech, EK282/4-96), and IL-1β (MultiSciences Biotech, EK201B/3-96) were detected by the ELISA kits following the manufacturer's protocols, whereas protein content was determined using the BCA protein assay kit.

### Histological assessment of lung injury

After anesthesia, the left lung lobe of each mouse was dissected and fixed in neutral formaldehyde. The paraffin wax-embedded sections were then flattened on slides, followed by hematoxylin-eosin (H&E) staining. Slides were imaged under a Nikon Eclipse C1 microscope (Nikon, Japan) and the degree of injury was assessed by 2 independent researchers in a blinded manner based on the international scoring system (16). Meanwhile, the slides were stained with the primary antibodies against myeloperoxidase (MPO) (Abcam, ab20867, UK) to determine neutrophil activation, and Oil Red O to quantify lipid/fatty acid contents in the lung. They were imaged under a microscope and analyzed using ImageJ software.

### Statistical analysis

All the statistical analyses were performed using GraphPad Prism software (USA) (version 8.0). Specifically, for the comparison between 2 groups, two-tailed unpaired Student’s *t*-test was applied, while for the comparison among multiple groups, one-way ANOVA followed by Bonferroni's *post hoc* test was utilized. The difference was identified as statistically significant when P<0.05. All the experiments were repeated independently at least 3 times. Quantitative data are reported as means±SE.

## Results

### Lipid accumulation in pulmonary macrophages increased in the LPS-induced ALI murine model

**Figure 1:**
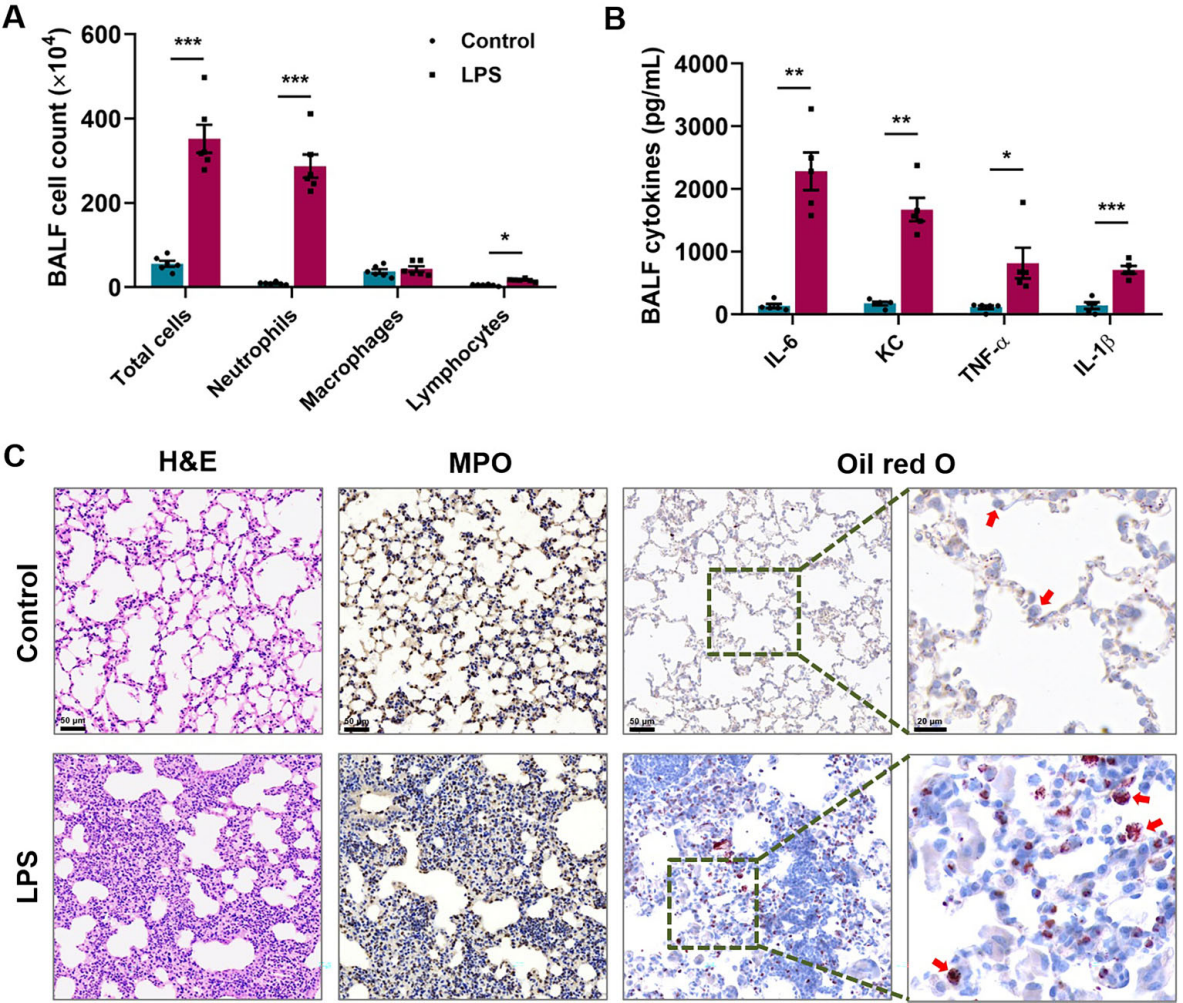
Lipid accumulation in pulmonary macrophages increased in a lipopolysaccharide (LPS)-induced acute lung injury (ALI) murine model. **A** and **B**, LPS administration dramatically increased total cell number and neutrophil and lymphocyte counts (**A**), as well as the chemokine keratinocyte chemoattractant (KC) and cytokines interleukin (IL)-6, tumor necrosis factor (TNF)-α, and IL-1β (**B**) levels in the mouse bronchoalveolar lavage fluid (BALF). **C**, Representative images of lung sections stained with HE (left panels), myeloperoxidase (MPO) (middle panels), and Oil Red O (right four panels) from mice with or without LPS challenge. Red arrows indicate macrophages. Scale bar in the left six panels: 50 μm; scale bar in the two far right panels: 20 μm. Data are reported as means±SE (n=5-6). *P<0.05, **P<0.01, ***P<0.001. Statistical analysis was performed using two-tailed unpaired Student *t*-test.

In this study, we adopted a classic ALI murine model induced by intranasal LPS administration. After 24 h of challenge, the lung inflammation and tissue injury were estimated comprehensively. As shown in Figure 1A, the counts of total inflammatory cells, neutrophils, and lymphocytes in BALF were significantly increased in ALI mice. LPS administration also elevated the levels of pro-inflammatory chemokine KC and cytokines IL-6, TNF-α, and IL-1β in the BALF (Figure 1B). In addition, histological features of tissue slides were used to evaluate the severity of lung damage. It was clearly observed that LPS exposure induced pulmonary injury, embodied by increased alveolar neutrophils, interstitial neutrophils, hyaline membranes, proteinaceous debris, and alveolar septal thickening (Figure 1C, left column). As a hallmark of ALI, enhanced MPO expression was also seen in neutrophils of ALI mice, which suggested significant neutrophil activation within lung tissues (Figure 1C, middle column). Moreover, the Oil Red O staining demonstrated that lipid accumulation was mainly distributed in inflammatory cells, especially macrophages (red arrows) of the ALI lung (Figure 1C, right columns). The disrupted lipid homeostasis caused by LPS indicated inhibited mitochondrial FAO in the macrophages of ALI mice.

### Lca dampened LPS-triggered inflammation in THP-1 derived macrophages

**Figure 2:**
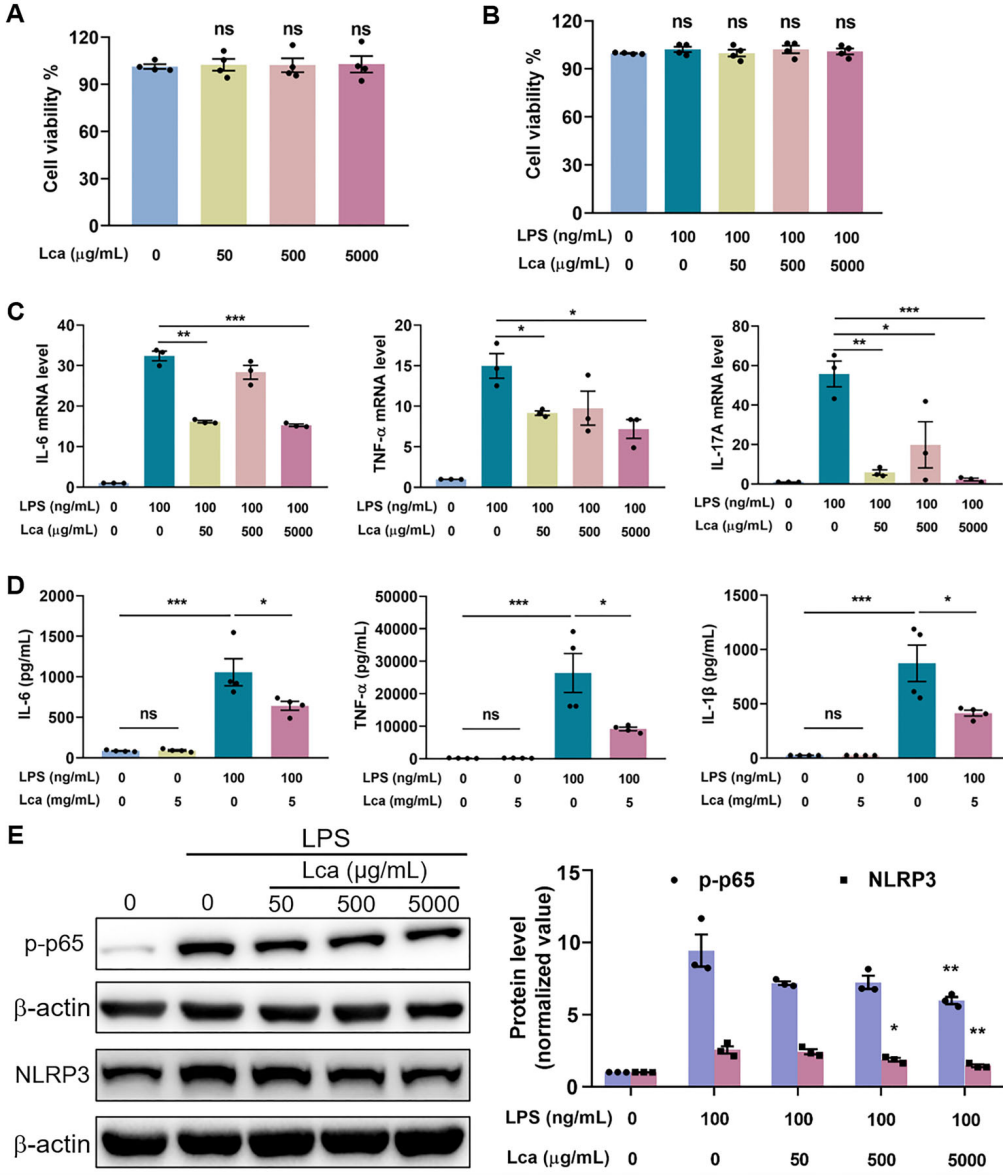
L-carnitine (Lca) suppressed lipopolysaccharide (LPS)-induced inflammation in THP-1-derived macrophages. **A** and **B**, Effects of Lca alone or in combination with LPS on cell viability of THP-1 cell-derived macrophages. **C** and **D**, Effects of Lca on LPS-elevated interleukin (IL)-6, tumor necrosis factor (TNF)-α, IL-17A mRNA (**C**), and IL-6, TNF-α, IL-1β secretion (**D**) in THP-1 cell-derived macrophages. **E**, Immunoblotting and related quantitative analysis of p-p65 and NLRP3 level for pro-inflammatory pathway activation in each group. Data are reported as means±SE (n=3-4). *P<0.05, **P<0.01, ***P<0.001, ns: not significant (**A**-**D**). *P<0.05, **P<0.01 *vs* LPS group (**E**). Statistical analysis was performed using one-way ANOVA followed by Bonferroni's *post hoc* test.

Based on the above data, we hypothesized that the disrupted FAO contributed to the excessive inflammation in LPS-exposed macrophages. Herein, we applied Lca, an activator for mitochondrial FAO, that could ameliorate lipid metabolic imbalances to modulate the inflammatory responses triggered by LPS in THP-1 cell-derived macrophages. Firstly, the cellular toxicity of Lca was estimated by CCK-8 assay. Compared with the control group, 5 mg/mL Lca with or without LPS stimulation did not affect the viability of macrophages (Figure 2A and B). Then, we found that Lca could down-regulate the mRNA expression of IL-6, TNF-α, and IL-17A (Figure 2C), as well as decrease the secretion of IL-6, TNF-α, and IL-1β (Figure 2D) in LPS-stimulated THP-1 cell-derived macrophages. Through the immunoblotting assay, we further determined that Lca suppressed LPS-induced activation of pro-inflammatory NF-κB/NLRP3 pathway (Figure 2E). Collectively, these results suggested that Lca possessed a potent anti-inflammatory activity *in vitro*.

### Lca maintained FAO and mitochondrial homeostasis under LPS stimulation in macrophages

**Figure 3:**
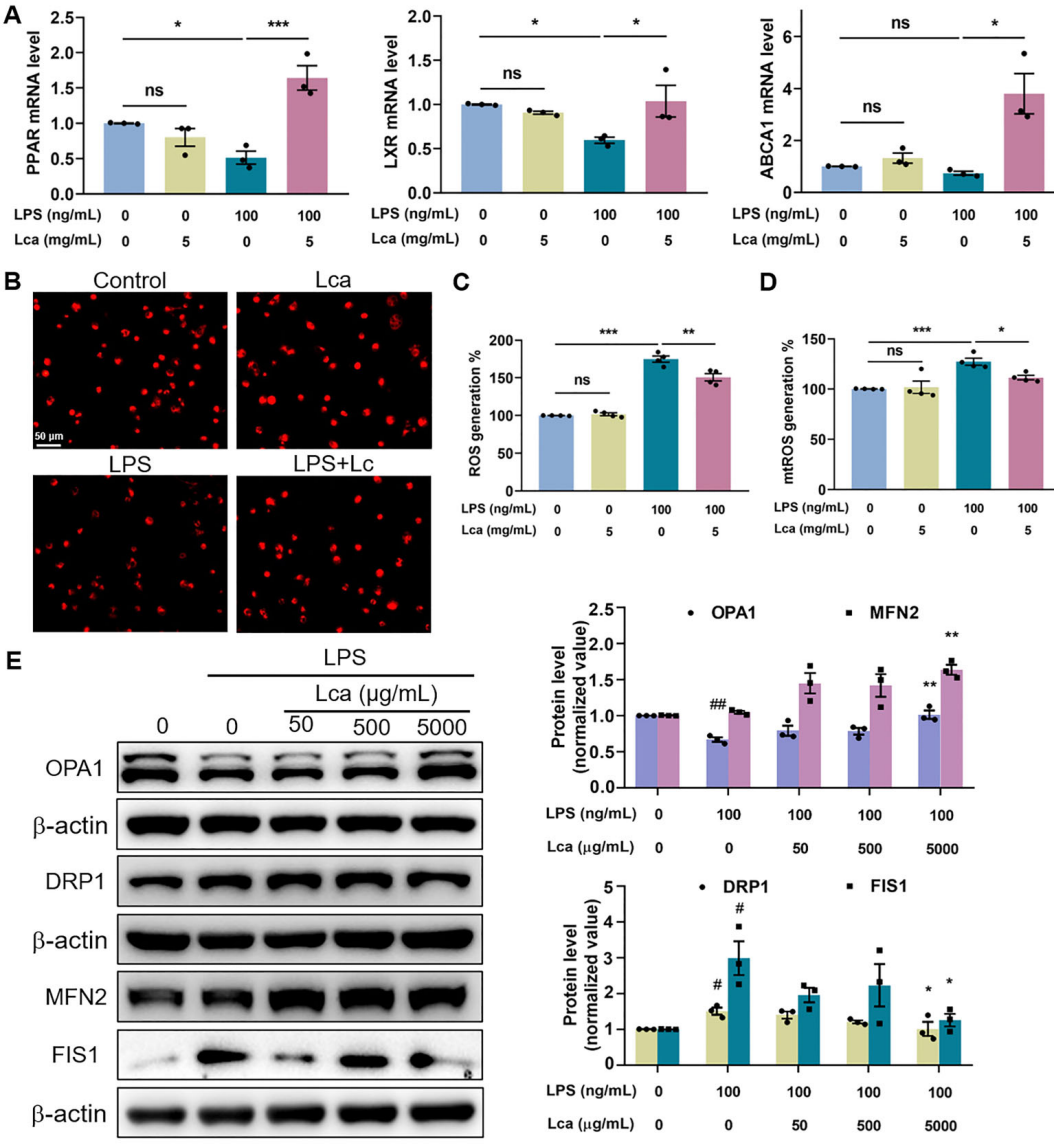
L-carnitine (Lca) maintained fatty acid oxidation (FAO) and mitochondrial homeostasis under lipopolysaccharide (LPS) stimulation in THP-1-derived macrophages. **A**, Effects of Lca on PPAR, LXR, and ABCA1 mRNA expression in macrophages upon LPS stimulation. **B**, Fluorescence images showing the impact of Lca on mitochondrial integrity (stained with MitoTracker Red probe, shown in red) in THP-1 derived macrophages. Scale bar: 50 μm. **C** and **D**, Impact of Lca on LPS-induced intracellular and mitochondrial (mt) reactive oxygen species (ROS) production in macrophages using DCFH-DA (**C**) and MitoSOX (**D**) fluorescence assay, respectively. **E**, Immunoblotting and related quantitative analysis of OPA1 and MFN2 for mitochondrial fusion, as well as DRP1 and FIS1 for mitochondrial fission in THP-1-derived macrophages. Data are reported as means±SE (n=3-4). *P<0.05, **P<0.01, ***P<0.001, ns: not significant (**A**-**D**). ^#^P<0.05, ^##^P<0.01 *vs* Control group; *P<0.05, **P<0.01 *vs* LPS group (**E**). Statistical analysis was performed using one-way ANOVA followed by Bonferroni's *post hoc* test.

In the process of lipid metabolism, several key factors such as PPAR, LXR, and ABCA1 participate in FAO to reduce lipid accumulation. Herein, we proved that LPS exposure inhibited the expression of PPAR and LXR, while Lca administration significantly increased the mRNA levels of PPAR, LXR, and ABCA1 (Figure 3A), indicating a critical role of Lca in maintaining FAO in macrophages upon LPS stimulation. Mitochondria are capital organelles in the regulation not only of energy but also of free radical metabolism. Mitochondria dysfunction has been demonstrated to participate in the pathogenesis of ALI/ARDS. Accordingly, we used MitoTracker fluorescent probe to assess mitochondrial activity in each group and visualized weaker fluorescence after LPS challenge, which could be elevated by Lca in THP-1 cell-derived macrophages (Figure 3B). Moreover, the effects of Lca on both intracellular and mitochondrial ROS generation were examined. As shown in Figure 3C, intracellular ROS was dramatically elevated upon LPS stimulation, whereas Lca pretreatment could reduce ROS production by use of the DCFH-DA fluorescent probe. For mitochondrial ROS detection, we employed a cell-permeable fluorescent indicator, MitoSox, and clearly found that macrophages pretreated with Lca produced less mitochondria-specific ROS in response to LPS than those without Lca treatment (Figure 3D). Notably, Lca alone had no effect on either intracellular or mitochondrial ROS compared with the control group (Figure 3C and D). In order to explore mechanisms underlying the regulation of Lca in mitochondrial ROS-scavenging activity, we further investigated mitochondrial dynamics upon LPS challenge. Herein, we found that Lca significantly enhanced the expression of mitochondrial fusion proteins OPA1 and MFN2, while it decreased the fission proteins DRP1 and FIS1 (Figure 3E) in response to LPS. Altogether, the results indicated that Lca was able to mitigate mitochondrial dysfunction, which might be attributed to the amelioration of mitochondrial dynamics in LPS-stimulated macrophages.

### Lca pretreatment decreased lung inflammation in the ALI murine model

**Figure 4:**
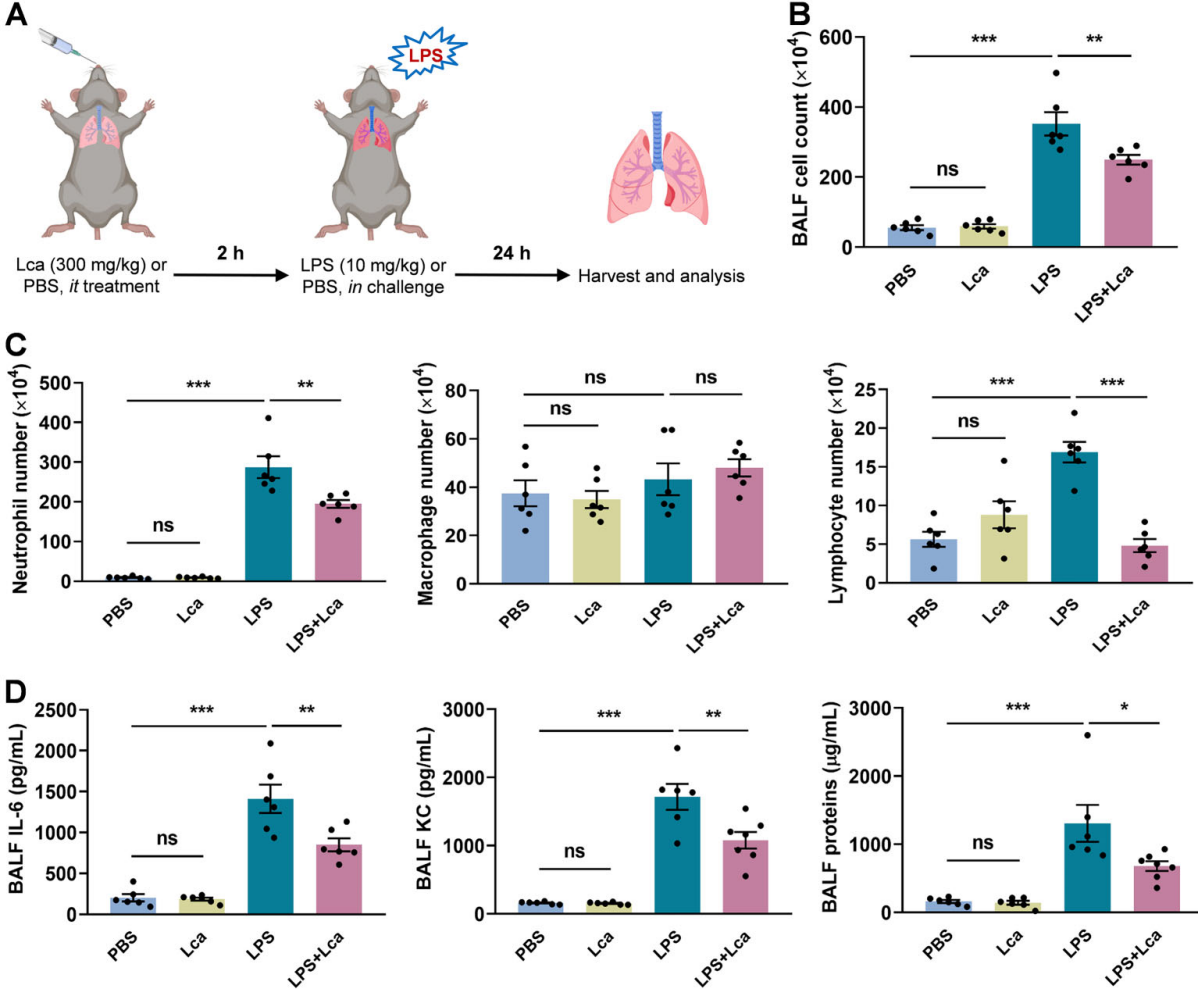
L-carnitine (Lca) pretreatment decreased lung inflammation in an acute lung injury (ALI) murine model. **A**, The lipopolysaccharide (LPS)-induced ALI murine model. **B**-**D**, Lca administration significantly reduced the numbers of total cells (**B**), especially neutrophils and lymphocytes (**C**), as well as cytokine interleukin (IL)-6, chemokine keratinocyte chemoattractant (KC), and total protein concentration (**D**) in bronchoalveolar lavage fluid of ALI mice. Data are reported as means±SE (n=6). *P<0.05, **P<0.01, ***P<0.001, ns: not significant. Statistical analysis was performed using one-way ANOVA followed by Bonferroni's *post hoc* test.

Since Lca exhibited potent regulatory activity on excessive inflammation and mitochondrial dysfunction induced by LPS in THP-1 cell-derived macrophages, we speculated that Lca had the potential to dampen the pulmonary inflammation in ALI/ARDS. To test the hypothesis, we investigated the efficacy of Lca in the LPS-induced ALI mouse model. Specifically, Lca (300 mg/kg) was administered intratracheally 2 h before intranasal LPS (10 mg/kg) challenge. After 24 h of exposure, BALF and lung samples were collected for further analyses (Figure 4A). We identified that ALI mice had elevated inflammatory cells in the BALF, while Lca pretreatment significantly inhibited the accumulation of total cell numbers, as well as neutrophils and lymphocytes (Figure 4B and C). In addition, the pro-inflammatory cytokine IL-6 and chemokine KC, and the total protein content in the BALF were also reduced by Lca administration (Figure 4D). Notably, Lca alone showed no effects on cell infiltration, cytokine secretion, and protein accumulation in the airway (Figure 4B and D). The findings supported that Lca was effective in preventing lung inflammation in ALI mice.

### Lca administration protected mice from tissue damage and mitochondrial dysfunction in the ALI model

**Figure 5:**
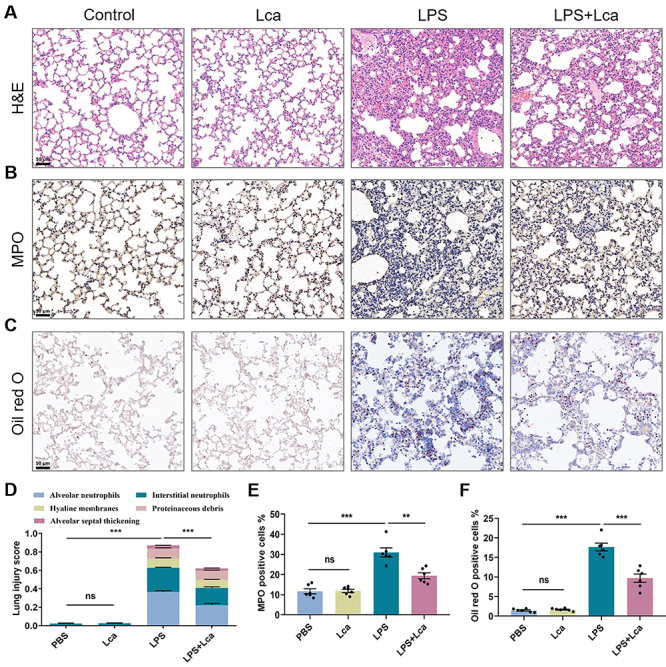
L-carnitine (Lca) protected mice from tissue damage in the lipopolysaccharide (LPS)-induced acute lung injury (ALI) model. **A** and **D**, Histological images and the related lung injury score of H&E-stained lung sections from distinct groups. **B** and **E**, Immunohistochemical images and quantitative analysis of MPO expression in lung sections from different groups. **C** and **F**, Representative images and quantitative analysis of lipid accumulation in murine lungs from each group. Scale bar: 50 μm. Data are reported as means±SE (n=6). **P<0.01, ***P<0.001, ns: not significant. Statistical analysis was performed using one-way ANOVA followed by Bonferroni's *post hoc* test.

**Figure 6:**
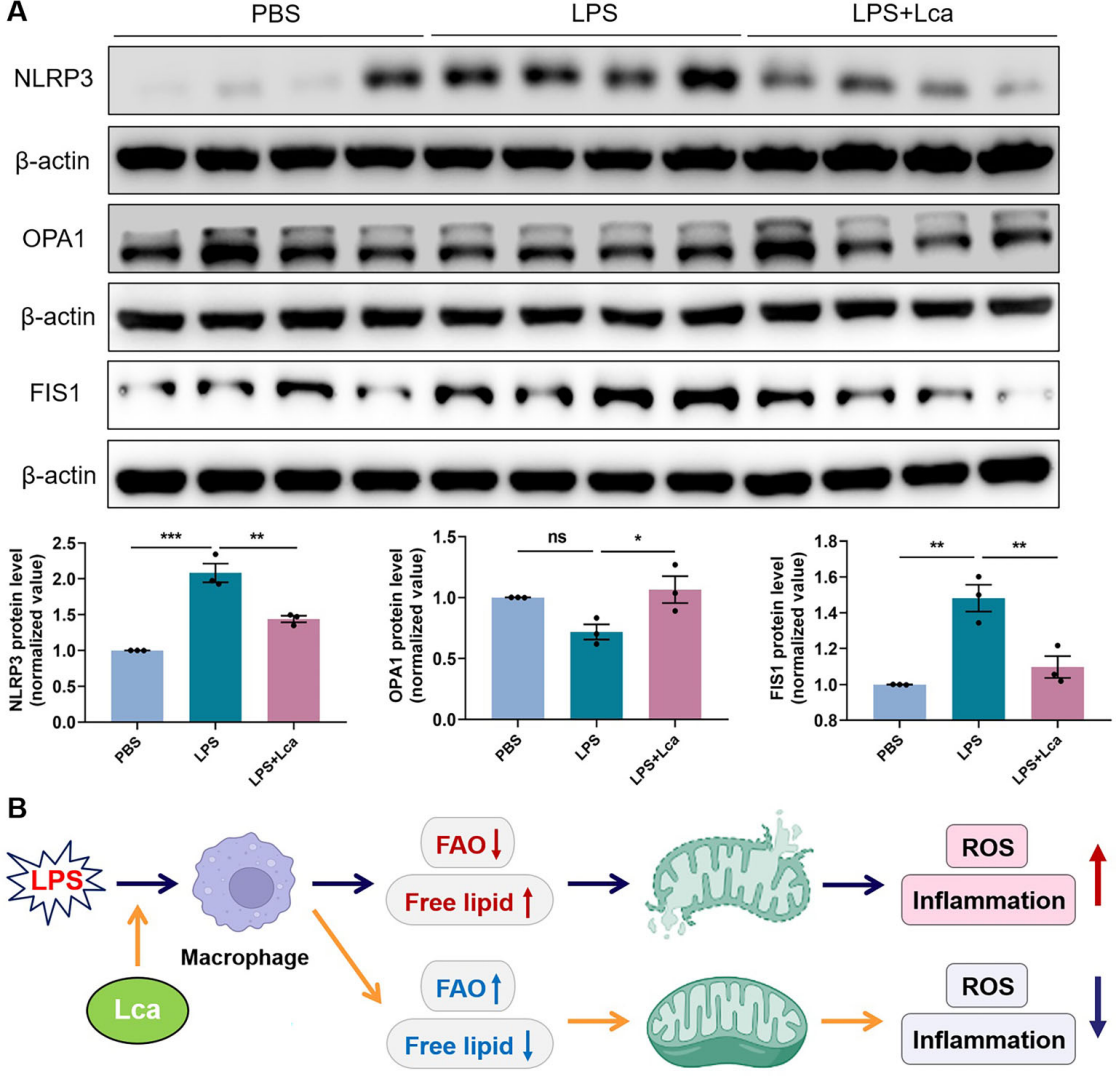
L-carnitine (Lca) administration improved mitochondrial dysfunction in lung tissues of lipopolysaccharide (LPS)-induced acute lung injury (ALI) mice. **A**, Immunoblotting and its quantitative analysis of NLRP3 expression for inflammation activation, OPA1 level for mitochondrial fusion, as well as FIS1 level for mitochondrial fission in mice lungs from distinct groups. **B**, Diagram of modulatory effects of Lca on pulmonary macrophages in LPS-induced ALI model. Data are reported as means±SE (n=3). *P<0.05, **P<0.01, ***P<0.001, ns: not significant. Statistical analysis was performed using one-way ANOVA followed by Bonferroni's *post hoc* test.

The degree of LPS-induced lung injury of ALI mice was evaluated by histopathological analysis following five features: alveolar neutrophils, interstitial neutrophils, hyaline membranes, proteinaceous debris, and alveolar septal thickening. As shown in Figure 5A and D, Lca pretreatment significantly decreased the total injury score in ALI mice. Considering that ALI is an acute inflammation dominated by neutrophils, we further assessed the neutrophil activation, marked by MPO expression. It was shown that lung sections of Lca-pretreated mice had less MPO staining compared with LPS challenge alone (Figure 5B and E). Moreover, Lca reduced free fatty acid accumulation in pulmonary inflammatory cells, indicating the enhanced mitochondrial FAO in the ALI model (Figure 5C and F). Highly consistent with the *in vitro* results, we identified that Lca restrained the expression of NLRP3 and FIS1, whereas it up-regulated the protein level of OPA1 in the whole lysates of ALI lung tissue (Figure 6A). Overall, we hypothesized that Lca treatment maintained intracellular lipid homeostasis, ameliorated mitochondrial dynamics, and decreased diffuse inflammatory damage in the LPS-challenged ALI mice.

## Discussion

The lung has rarely been seen as a lipid metabolic organ in the past. However, in the alveolar areas, lipid metabolism is carried out actively to modulate surfactant homeostasis and ensure normal respiratory cycle. Disrupted lipid metabolism induces mitochondrial FAO inhibition, excessive inflammation, and ROS overproduction, thus leading to the pathogenesis of various pulmonary diseases. For example, impaired FAO in alveolar epithelial cells contributed to the overwhelming inflammation and diffuse damage in an ALI murine model (7). Lca is the key metabolite during the transport of long-chain free fatty acids into the mitochondria for subsequent FAO. In a cigarette smoke-induced cellular model of emphysema, FAO inhibition elevated the apoptosis of lung endothelial cells; conversely, enhanced FAO by Lca was beneficial to the pathological process (17). Additionally, Lca also showed protective efficacy in an animal model of pulmonary emphysema triggered by elastase (18). In the lung injury model of newborn mice mediated by hyperoxia, Lca treatment promoted FAO, therefore reducing the tissue injury in neonates (8). Nevertheless, there were still no studies on the effects of Lca in the LPS-induced ALI murine model. Herein, we discovered disturbed lipid metabolism in inflammatory cells, especially macrophages infiltrated into the lung and demonstrated that Lca pretreatment effectively mitigated inflammatory damage in ALI mice. Furthermore, Lca effectively modulated inflammatory response of THP-1 cell-derived macrophages in *in vitro* experiments. These findings provided us with evidence that Lca could be a novel and promising therapy for ALI/ARDS.

Mitochondria are bilayer-membrane organelles in cytoplasm of most eukaryotic cells. They are often considered powerhouses, where various metabolic reactions proceed efficiently, represented by glucose metabolism and lipid metabolism. Because multiple substrates and catalytic enzymes are located in the mitochondrial membranes, the disturbance of energy metabolism could induce mitochondrial impairment. For instance, increased glycolysis prevented airway epithelial cells from mitochondrial dysfunction and acute injury (19). Moreover, impaired FAO might lead to impaired mitochondrial bioenergenesis in alveolar epithelial cells (7). In the present study, we validated that Lca treatment up-regulated the expressions of PPAR, LXR, and ABCA1, which participate in FAO and maintain lipid homeostasis (20), in macrophages upon LPS stimulation. Additionally, Lca exhibited a strong mitochondrial-protecting effect in THP-1-derived macrophages. Specifically, Lca elevated mitochondrial activity and mitochondrial ROS-scavenging capacity under LPS stimulation. Simultaneously, Lca treatment modulated the expression of mitochondrial fusion proteins OPA1 and MFN2, and mitochondrial fission proteins DRP1 and FIS1, thus ameliorating mitochondrial dynamics in LPS-challenged macrophages. As mitochondria dysfunction has been demonstrated to participate in inflammatory over-reaction and ROS over-production during the pathogenesis of various diseases like ALI/ARDS (21,22), we speculated that the study not only expanded our understanding of ALI pathogenesis, but also broadened the clinical applications of existing drugs for different diseases.

Intensive study has identified the pivotal role of macrophages in the pathogenesis of ALI/ARDS (1,3). In the initial stage of the syndrome, macrophages are activated quickly to recognize and eliminate the invading pathogens. However, over-activated macrophages may drive detrimental inflammation and pulmonary damage via secreting large amounts of cytokines and attracting many immune cells (including circulating neutrophils, macrophages, and lymphocytes) to the lung. Therefore, controlling the overwhelming inflammatory responses of macrophages in the early stage may become a promising strategy to treat ALI/ARDS. With the study of the novel mechanisms underlying the inflammation of lung macrophages, lipid metabolism has been reported to participate in the pathophysiological process. Soliman et al. (9) reported that defective FAO in chronic MWCNT-exposed alveolar macrophages resulted in excessive oxidative stress and pulmonary granulomatous inflammation in a murine model. In the current study, we demonstrated that Lca reduced free fatty acid accumulation in alveolar macrophages, which might effectively suppress the excess inflammation in LPS-induced ALI mice. In THP-1-derived macrophages, Lca dramatically increased the expression of PPAR, LXR, and ABCA1 upon LPS challenge, which was consistent with the findings that activation of the PPAR-LXR-ABCA1 pathway achieved an anti-inflammatory effect, and ligands of PPARs caused a substantial reduction of experimental ALI (23,24). Furthermore, Lca inhibited LPS-elevated expression of pro-inflammatory cytokines IL-6, TNF-α, IL-17A, and IL-1β in macrophages and IL-6 and KC in the BALF of ALI mice, which were all related to the formation and development of ALI/ARDS (25,26).

The present study had some limitations. Firstly, the work was performed only in the THP-1 cell line, and primary cells such as bone marrow-derived macrophages and other immune cell types should be used in future studies. Secondly, several protective actions of Lca have been determined, whereas the underlying molecular mechanisms are not fully understood. Whether the up-regulation of key enzymes for FAO alleviates ALI in other disease models should also be investigated in future studies.

### Conclusion

In summary, Lca, the mitochondrial FAO activator, showed potent anti-inflammatory effect on THP-1-derived macrophages upon LPS stimulation. Mechanistically, Lca maintained mitochondrial activity and ameliorated mitochondrial dynamics (Figure 6B). In the LPS-induced ALI mice, Lca inhibited neutrophilic inflammation and decreased diffuse tissue damage, which might be due to the preservation of mitochondrial homeostasis. These data broadened our understanding of ALI/ARDS pathogenesis and provided a promising drug candidate for this syndrome.
